# Interactivity in learning instructional videos: Sending danmaku improved parasocial interaction but reduced learning performance

**DOI:** 10.3389/fpsyg.2022.1066164

**Published:** 2022-12-12

**Authors:** Ya Mou, Bin Jing, Yichun Li, Nanyang Fang, Changcheng Wu

**Affiliations:** ^1^Faculty of Artificial Intelligence in Education, Central China Normal University, Wuhan, Hubei, China; ^2^Shuang Liu Middle School, Chengdu, Sichuan, China; ^3^School of Computer Science, Sichuan Normal University, Chengdu, Sichuan, China; ^4^Qingtai Mountain Middle School Attached to Sichuan Normal University, Chengdu, Sichuan, China

**Keywords:** instructional video, danmaku, learning performance, cognitive load, parasocial interaction

## Abstract

**Introduction:**

The instructional video is considered to be one of the most distinct and effective virtual learning tools. However, one of its biggest drawbacks is the lack of social interaction that occurs. This study tested the impact of participants sending zero danmaku (sending messages on the screen), three danmaku sending, and unlimited danmaku as an instructional video plays on learning performance.

**Methods:**

We assessed learners’ retention and transfer scores, as well as self-report scores for cognitive load and parasocial interaction. This study sample comprised 104 participants who were randomly assigned to learn from one of three instructional videos on the topic of the heart.

**Results:**

The results showed that sending danmaku improved learners’ parasocial interaction, while significantly increasing their cognitive load and also hindering learning performance. The observed increase in cognitive load reported by learners was also caused by increased levels of parasocial interaction.

**Discussion:**

Our findings suggest that by sending danmaku, learners can promote interactive learning, but that this has a negative impact on learning performance and the process of video learning.

## Introduction

The instructional video is considered to be one of the most distinct and effective virtual learning tools as it provides a sensory learning environment that has been shown to support learners to understand more and help them better recall knowledge (Palaigeorgiou and Papadopoulou, 2019). Furthermore, instructional videos also appear to have a significant effect on improving learners’ learning performance, engagement, interest, motivation to learn new subjects, and autonomy (Giannakos et al., 2015). However, instructional videos are not a panacea. Linear instructional videos run the risk of becoming a passive, television-like experience for learners. This type of experience can lead to superficial learning or inadequacy of the overall learning performance, also known as the “couch-potato” phenomenon (Van den Bulck, 2000; Palaigeorgiou and Papadopoulou, 2019). One of the most notable weaknesses of instructional videos is that learners cannot interact with it, so instructional videos often lack cognitive appeal and challenges (Palaigeorgiou and Papadopoulou, 2019). This interaction has been considered to be an important factor in improving social presence and learners’ attention (Rice and Redcay, 2016; Yang et al., 2019). In recent years, the production technology of instructional videos has grown rapidly, enabling more interactive functions, such as online forums or real-time in-video text messages, known as danmaku. Danmaku, as an emerging video interaction technology, allows users’ comments to be posted and displayed instantly (anonymously) on the video screen, thus becoming an integral part of the video; every time the video is played, it also plays all past comments (Zhou and Zhou, 2022). Danmaku were initially popular in China, Japan, and other Asian countries, and became sought after and loved by the majority of video-based learners (Teng and Chan, 2022). Especially in China, when learners are viewing a video, the playback platform typically has the “danmaku display” function turned on by default, allowing people to interact with other users while watching in text form (Bai et al., 2021). While such interactions are commonplace, little is known about the effects of seeing others’ danmaku in such videos on users’ attention and experience.

Most existing research on danmaku has been from the perspective of “learners seeing danmaku sent by others on the screen” (e.g., Pi et al., 2020), focusing on the potential value of the danmaku itself and their impact on learners’ attention as they watch the video lectures (Bai et al., 2019; Pi et al., 2020). For instance, learners’ interaction with danmaku-enabled videos can not only provide immediate feedback to video creators on how they might improve the quality of the video, but they also greatly enhance the viewing experience of online learners (Bai et al., 2021). Zhang et al. (2019) discussed the potential of danmaku and proposed to include them in their instructional videos, whether the content is synchronous or asynchronous. With danmaku, even an asynchronous experience can seem like a synchronous experience. It is a potentially useful tool for use in online learning as learners feel they are learning alongside peers and teachers (Zhang et al., 2019). However, Pi et al. (2020) showed through empirical research that seeing others’ danmaku during instructional videos had a negative impact on learners’ attention and learning.

Compared to seeing others’ danmaku onscreen, the biggest difference for learners in sending their own danmaku is interactivity (Ma and Cao, 2017; Pi et al., 2020). Learners communicate synchronously or asynchronously with others by sending danmaku. Thus, learners can have a “pseudo-synchronic” communication and viewing experience by replying to others’ danmaku comments, creating a sense of a “live” experience through the sense of shared virtual time between learners and learners, as well as between learners and instructors (Johnson, 2013). This “pseudo-synchronicity” not only changes the way learners experience instructional videos from a passive and isolated experience to a more active and social experience, but it also provides them with a sense of a shared and synchronous experience. Research has shown that this type of interaction appears to give learners an instant experience of immersion, similar to if they were having a conversation directly with other learners, experiencing a “mental sensation of engagement,” also known as parasocial interaction (Shin, 2019; Lim et al., 2020; Yang et al., 2022). To our knowledge, few studies have explored how learners actively sending danmaku affects their learning process and performance. Theoretically, the benefits of sending danmaku during the learning experience are consistent with the theory of social constructivism (Vygotsky and Cole, 1978). This theory proposes that learners are not only passive receivers of knowledge and information, but also active generators and constructors of knowledge. The creation of knowledge requires active interaction in a social environment. Social interaction must then exist prior to the process of learners internalizing knowledge, and as such, social interaction is essential for the creation of new knowledge, and interaction with others is conducive to learners’ learning in the zone of proximal development (Vygotsky and Cole, 1978). Specifically, as a tool to reflect social interaction in the learning process, danmaku-sending provides scaffolding for learners and helps them to reach their potential level of development. The basis of social interaction is the exchange of messages (Vygotsky and Cole, 1978; Pi et al., 2019). Numerous studies on instructional video-based learning have shown that learners benefit from exchanging messages regarding the information shared through the videos (Li et al., 2014; Hung and Chen, 2018). Real-time exchange of messages in the classroom when learners are able to pause videos has been shown to have a positive impact on attention and learning performance (Weisz et al., 2007; Li et al., 2014). When learners send danmaku, they also exchange messages within the videos, which we infer would promote deeper processing and internalization of knowledge.

Although the benefit of exchanging messages has been demonstrated under particular conditions, not all instructional video formats have yet been tested (Pi et al., 2020). In previous studies, learners sent messages after learning from instructional videos, rather than exchanging messages simultaneously during learning (i.e., seeing and sending danmaku; Weisz et al., 2007; Li et al., 2014). Unfortunately, in a danmaku-enabled video, the real-time exchange of messages does appear to negatively impact learning (e.g., Pi et al., 2020). Several reasons have been suggested for this. First, learners must pay attention to danmaku content while also following and understanding the learning content. This phenomenon tends to make learners lose themselves in receiving and responding to danmaku comments, thus neglecting to absorb and process the learning content itself, resulting in excessive cognitive load (Yang et al., 2019). This situation is likely to trigger a split-attention effect, in which the learner must mentally integrate information from multiple sources simultaneously (Ayres and Sweller, 2005). Second, the danmaku sent by fellow learners may also have nothing to do with video learning knowledge, and may also be negative. This also increases learners’ cognitive load and is not conducive to learning (Yang et al., 2019). Furthermore, an earlier study showed that creativity is inhibited if learners must pay attention to constant interactions with others (Diehl and Stroebe, 1987). In the case of instructional videos, this would apply to learners sending danmaku (Herrmann et al., 2013; Pi et al., 2020).

To sum up, in previous studies, learners have generally only played the role of a “bystander” in the exploration of the effects of danmaku during learning. Existing research has suggested that danmaku can be helpful in instructional videos (Yang et al., 2019), but it is important to understand how to further improve the quality of instructional video learning. Therefore, the current study investigated whether the sending and not sending of danmaku brings about differences in learning from instructional videos, and whether differences in the number of danmaku sent might optimize the effectiveness of video learning in terms of learning performance (i.e., retention and transfer), self-reported cognitive load (i.e., intrinsic, extraneous, and germane cognitive loads), and parasocial interaction after the instructional video is over.

Participants were asked to watch instructional videos on the subject of the heart. In the control condition, learners only watched the instructional videos and were not able to send danmaku (No Danmaku-Sending group; NDS). Under the experimental conditions, learners viewed the instructional video, with the difference between the experimental conditions being only the number of danmaku sent by the learners themselves, specifically: (1) the number of danmaku sent during the video was set at three (Three Danmaku Sent group; TDS), and (2) the number of danmaku sent was unlimited, whereby the learner was required to send more than three danmaku during the video (Unlimited Danmaku Sent group; UDS). For the TDS group, we found through the pre-experiment test, when the number of danmaku learners could send was allowed to be unlimited, that participants would send an average of three danmaku during the process of learning from a video. In the actual experiment, each participant in the TDS group sent the same number of danmaku (i.e., three), which facilitated scientific observation and analysis by fixing the experimental conditions.

We wanted to verify whether learning was affected by the number of danmaku sent. Therefore, based on the above literature review, this study proposed the following hypotheses:

**Hypothesis 1:** Among the three instructional video conditions, learners in the NDS group will have the best retention and transfer scores, followed by the TDS group, and finally by the UDS group.

**Hypothesis 2:** In the UDS condition, learners will report having the highest cognitive load (including intrinsic, extraneous, and germane cognitive load), followed by those in the TDS condition, and finally by those in the NDS condition.

**Hypothesis 3:** Learning from instructional video under the UDS condition, learners will report the highest level of parasocial interaction, followed by learners in the TDS condition, and finally by learners in the NDS condition.

**Hypothesis 4:** The number of danmaku sent by learners will be negatively associated with learning performance, and this relationship is mediated by parasocial interaction and cognitive load.

## Materials and methods

### Participants and design

The participants in the current study were 104 grade 10 high school students (64 females; age: *M* = 15.45, SD = 0.54) recruited from one high school in Chengdu, Sichuan, China, and were selected randomly from four classes. None of the participants had been taught in the school setting about the heart. The students were informed about the purpose and safeguards of the experiment and written informed consent was obtained prior to participation. All participants were native Chinese and naive to this study. The study was approved by the ethics committee of the school where the study was conducted, and the study followed ethical standards of care for human subjects.

A one-factor between-subjects design was adopted in the present study with students assigned randomly to one of the three groups: 35 students (22 females; age: *M* = 15.43, SD = 0.56) to the NDS group, 35 students (24 females; age: *M* = 15.46, SD = 0.56) to the TDS group, and 34 students (18 females; age: *M* = 15.47, SD = 0.51) to the UDS group. There were no significant differences between these three groups in mean age [*F*(2,101) = 0.05, *p* = 0.947], their mean pre-test scores [*F*(2,101) = 0.15, *p* = 0.858], or on the proportion of males and females, χ^2^(2) = 1.82, *p* = 0.403.

### Materials

The instructional video ran for 6 min and 18 s, and the content was on “Heart and blood circulation.” We used PowerPoint and Adobe Premiere to create the video. The instructional video consisted of three parts: danmaku, learning content, and voice. Regarding the danmaku, when we were making the video, we use the “four classes of communicative approach” to design four kinds of questions (Scott et al., 2006). These questions were used to create the pseudo-danmaku that would be used to answer the questions. In this experiment, we designed a total of 30 pseudo-danmaku. These danmaku ran from the right of the screen to the left at a consistent speed, positioned in the top fifth of the screen above the video and scrolling past until they disappeared. The questions were also intended to guide learners to send their own danmaku in response to the questions raised in the videos. Some of the questions used are shown in Table 1. Regarding the learning content, the instructional video was made up of 12 pages of slides, with the key instructing content presented in the form of text, accompanied by an illustration to help learners understand the content. Finally, regarding the voice, the audio content was recorded by a male instructor with a neutral Chinese accent. All materials went through multiple rounds of screening and revision by two master students before they were combined to create the final instructional video.

**TABLE 1:** Specific questions used to encourage social interaction: four classes of communicative approach.

Number	Type	Sample question
1	Dialogic-interactive	*Q:* Do you know which two types of cells the cardiac muscle contain? *A:* Yes, the cardiac muscle cells contain cardiomyocytes and special cardiomyocytes.
2	Non-dialogic-interactive	*Q:* Coronary veins can also provide blood for the heart. Do you know the difference between coronary veins and arteries? *A:* Yes, the coronary veins can also provide blood for the heart.
3	Dialogic-non-interactive	The heart is a complex organ, we should not only learn the different locations of its parts but also the function of each part. For example, this is the part that keeps the heart beating, and this is the part that supplies the blood.
4	Non-dialogic-non-interactive	The cardiac muscle is the part of the heart that we normally see, and it makes up the outline of our heart.

**FIGURE 1:**
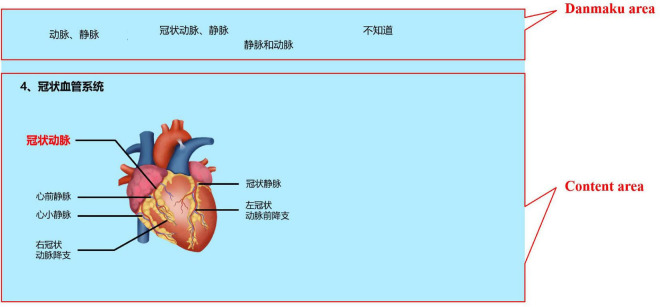
Danmaku and video content positioning on instructional video screen in Dandan Play player.

The video was shown using software called “Dandan Play.”^1^ This software allows learners to send danmaku in the instructional videos over a local area network without having to register and account. The software also supports changing the color, size, and screen location of the danmaku. Using intelligent identification of video content, past danmaku saved on the server can be synchronized to appear in the video on the local computer to enhance the viewing experience. The danmaku are shown in the top fifth of the screen, while the rest of the screen is taken up by the video content (see Figure 1).

### Measurements

#### Pre-questionnaire

The pre-questionnaire consisted of three parts: first, demographic information (e.g., gender and age) was collected. Second, contextualizing danmaku in learners’ experience, both viewing (e.g., “If there are danmaku in the instructional video, do you think the danmaku will improve or interfere with your learning ability? Why?”) and sending (e.g., “If you send danmaku while learning from the instructional video, do you think this behavior will improve or interfere with your learning? Why?”). Finally, learners’ general knowledge about the heart was also tested, using seven multiple-choice items. An example item was: “Which of the following is not part of the blood circulatory system? A. Blood; B. Blood vessel; C. Heart; D. Lung; E. I don’t know.” Each item had five choices, and only one was the correct answer. The fifth option was “I don’t know” to avoid participants simply guessing the answer (Pi et al., 2022). Two points were awarded for each correct answer. The total score could range from 0 to 14. As there were relatively fewer items used in this study than in previous studies, the reliability analysis was measured using Spearman–Brown reliability (Kelley, 1925). The prior knowledge test showed moderate internal consistency: *r*_*kk*_ = 0.63 (LeBreton and Senter, 2008). The learners’ age, gender, and prior knowledge scores were used to determine whether the groups differed in their basic characteristics. These variables did not have any significant differences among the three groups.

#### Learning performance

Tests were created to assess learning performance, in terms of both knowledge retention and transfer. The knowledge retention test evaluated learners’ direct memory of knowledge after they had finished watching the learning instructional video. The retention test showed moderate internal consistency: *r*_*kk*_ = 0.60 (LeBreton and Senter, 2008). It contained 12 items with a possible total of 30 points. There were eight multiple-choice items with only one correct response (e.g., “Which of the following statements about the function of the human heart is false? A. The heart provides adequate blood flow to the body’s organs and tissues; B. The heart supplies oxygen to the body; C. The heart provides various nutrients to the body; D. The heart can bring carbon dioxide, urea, and so on to the cells; E. I don’t know.”), two multiple-choice items with several correct responses (e.g., “Which of the following statements about the heart are correct? A. The heart is positioned with the lungs on both sides; B. Above the heart is the diaphragm and below the heart are the large blood vessels that enter and exit the heart; C. The heart is located inside the middle mediastinum; D. The heart is located inside the posterior mediastinum; E. I don’t know.”), and two short-answer items regarding the coronary artery. Respondents received two points for each correct answer for the single-correct-choice items, three for the several-correct-choice items, and four points for short-answer items. The short-answer responses were scored separately by two raters and showed appropriate rater agreement (*r*s > 0.60, *p*s < 0.001, based on Pearson’s correlation).

The knowledge transfer test evaluated learners’ ability to use the presented material in novel situations after learning from the instructional videos. The transfer test had moderate internal consistency: *r*_*kk*_ = 0.59 (LeBreton and Senter, 2008). It contained eight multiple-choice items with one single correct response (e.g., “What happens when the coronary arteries in the human body harden? A. People get coronary heart disease; B. There is an embolism in the coronary arteries; C. There is a spasm in the coronary arteries; D. The cardiac muscle cannot get nutrients and oxygen; E. I don’t know.”), two multiple-choice items with several correct responses (e.g., “Which of the following symptoms of myocardial infarction are correct? A. Insufficient blood supply to the venous blood vessels; B. Decreased elasticity of the walls of the large arteries; C. A blockage in the terminal arteries of the heart; D. Necrosis in the area of coronary arteries; E. I don’t know.”), and a short-answer item about heart disease. The points for each item were set the same as those of the knowledge retention test (with eight points for the short-answer item) for a possible maximum of 30 points. Short-answer responses were scored separately by two raters and showed appropriate rater agreement (*r* = 0.67, *p* < 0.001, based on Pearson’s correlation).

#### Cognitive load

The cognitive load questionnaire asked learners to rate three subjective items intended to measure their perceptions concerning their intrinsic cognitive load (i.e., “How much effort was required to learn the material?”), extraneous cognitive load (i.e., “How difficult was the material?”), and germane cognitive load (i.e., “Did you think the material was easy to learn?”) during learning. The cognitive load items have been shown to be reliable and valid indications of cognitive load in previous studies (Moreno et al., 2001; Yung and Paas, 2015). The first two items were adapted from Craig and Schroeder (2017) and translated into Chinese by Yang (2014). The last item was adapted from Gerjets et al. (2009) and translated into Chinese by Zhao (2014). Learners rated each item on a nine-point scale ranging from 1 (very little) to 9 (very much).

#### Parasocial interaction

The parasocial interaction questionnaire was developed by Beege et al. (2017) and is used to test learners’ perception of the conversational “give and take” with the instructor. It consists of seven items (e.g., “I feel like the instructor is giving me a lesson”) and each item is rated using a seven-point Likert scale ranging from 1 (I do not agree at all) to 7 (I totally agree). The final score is the average of all item scores. To adapt the questionnaire for use in a Chinese context, all items were translated into Chinese and revised by a professional college English instructor (Pi et al., 2021). Cronbach’s alpha for the questionnaire in the current study was 0.94.

### Procedure

This study was conducted in a computer room and took approximately 45 min to complete. Before starting the experiment, each computer was checked to ensure that the headphones were plugged and the volume was adjusted to avoid interference from external conditions. The make and model of all computers and monitor screen sizes were identical. All computers were uniformly controlled by the researcher’s aide, and the learners wore headphones to avoid interfering with one another. When the experiment began, learners were first assigned randomly to the NDS, TDS, or UDS group, and each individual was told they would perform the tasks individually. The learners were then asked to sit in front of the computer and to complete the pre-questionnaire at their own pace. Next, the learners watched the instructional video for their specific group, and afterward completed the retention and transfer tests without having to adhere to a time limit. Finally, they were asked to complete a post-questionnaire to measure their perception of the learning process. After the experiment, all learners were compensated in the form of small gifts (i.e., facial tissue and pen) for their participation to thank them for their time and effort.

## Results

**FIGURE 2:**
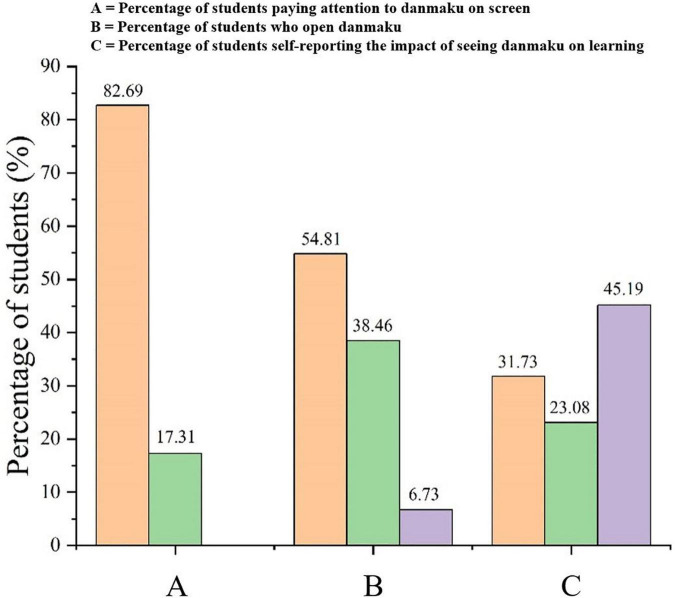
Learner expectations regarding the impact of viewing danmaku during instructional videos. **(A)** Differences in intrinsic cognitive load across experimental conditions; **(B)** differences in extraneous cognitive load across experimental conditions; and **(C)** differences in cognitive load across experimental condition.

Figure 2 depicts the learners’ screen setup when seeing danmaku in the instructional videos. As shown in Figure 2A, 82.69% of the learners indicated that they had paid attention to the danmaku sent by other learners while viewing the instructional video. This percentage was significantly higher than those who reported “not paying attention” (17.31%). Figure 2B shows that more than half of the learners (54.81%) reported that they would open the danmaku while the instructional video played, which was slightly higher than those who reported “closed the danmaku” (38.46%), and only 6.73% of the learners reported not paying any attention to the danmaku. Figure 2C shows that nearly half of the learners (45.19%) believed that the danmaku had no influence on their learning, while the proportion of learners who believed that the danmaku would improve their learning (31.73%) or that they would interfere with their learning (23.08%) were similar. The reasons suggested as to why the danmaku might improve their learning ability were that danmaku can supplement knowledge points, they present an opportunity for communication and discussion with others, and danmaku would expand the learner’s own thinking abilities. However, the reasons why danmaku might interfere with learning were that the danmaku would block the screen and distract them.

**FIGURE 3:**
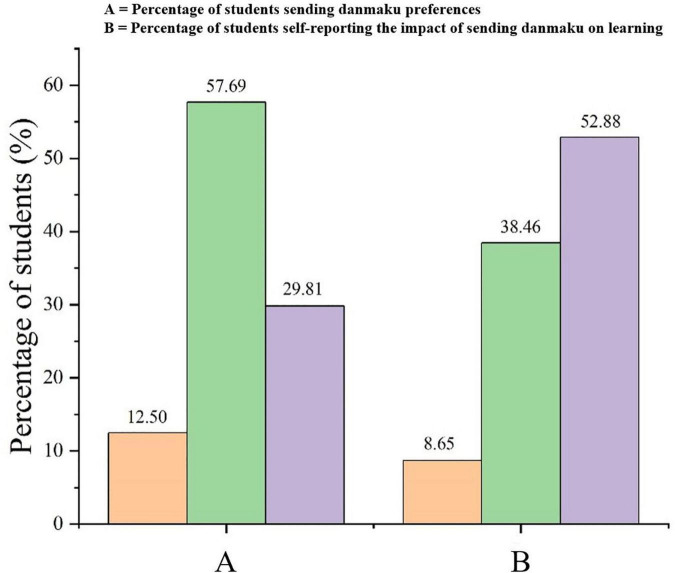
Learner expectations regarding the impact of sending danmaku during instructional videos. **(A)** Differences in retention across experimental conditions and **(B)** differences in transfer across experimental conditions.

Figure 3 presents the preference of learners sending danmaku in the instructional videos. Figure 3A showed that most learners (57.69%) did not like sending danmaku during the learning process; a small number of learners (29.81%) did not have any preference about sending danmaku; while only 12.50% of learners enjoyed sending danmaku. Only a very small percentage of learners (8.65%) believed that sending danmaku would improve their learning, reporting that sending danmaku would be a way to communicate with others, which would help them participate more actively in class interactions, enabling them to understand the problems and solve them quicker. In contrast, a significant number of the learners (38.46%) believed that sending danmaku would hinder their learning (see Figure 3B), thinking that it would be a waste of time or that it would distract them. Finally, 52.88% of the learners thought that sending danmaku would have no impact on their learning.

Descriptive statistics for all variables in the three groups are reported in Table 2. To test the differences across the different groups, one-way ANOVA was conducted for all variables using SPSS 24.0. Mplus 8.3 was used to analyze the mediating effect [bootstrap analysis, repeat sampling, 2,000 times following recommendations by DiCiccio and Efron (1996)]. In the current research, statistical significance was set at *p* < 0.05. In ANOVA, the effect size was represented by $\eta_{p}^{2}$; an $\eta_{p}^{2}$ of 0.01 indicated a small effect size, an $\eta_{p}^{2}$ of 0.06 indicated a moderate effect size, and an $\eta_{p}^{2}$ of 0.14 indicated a large effect size (Cohen, 2013).

**TABLE 2:** Means and standard deviations of all variables by group.

Dependent variables	NDS		TDS		UDS				
			
	*M*	SD	*M*	SD	*M*	SD	*F*	*p*	$\eta_{p}^{2}$
**Learning performance**									
Retention	18.84	4.07	15.79	3.21	13.50	3.40	19.37***	<0.001	0.28
Transfer	17.10	4.60	14.96	3.33	13.37	4.66	6.75**	0.002	0.12
Cognitive load	5.76	0.92	6.08	1.38	6.97	1.40	8.60***	<0.001	0.15
Intrinsic cognitive load	6.51	1.69	7.06	1.63	8.15	1.33	9.81***	<0.001	0.16
Extraneous cognitive load	4.43	1.80	4.57	1.98	6.09	2.04	7.72**	0.001	0.13
Germane cognitive load	6.34	1.83	6.60	2.35	6.68	2.24	0.23	0.796	0.01
Parasocial interaction	3.89	0.99	4.20	1.38	4.84	1.37	5.14**	0.007	0.09

NDS, no danmaku sent; TDS, three danmaku sent; UDS, unlimited danmaku sent; *M*, mean; SD, standard deviation; *F*, *F*-statistic; $\eta_{p}^{2}$, effect size. **p < 0.01, ***p < 0.001.

### Learning performance

**FIGURE 4:**
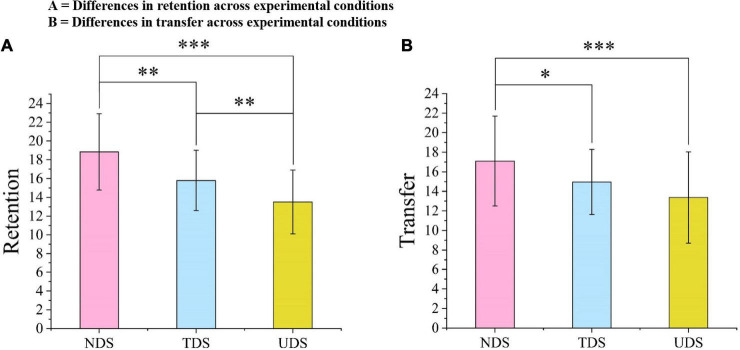
Differences in retention and transfer across experimental conditions. **(A)** Differences in retention across experimental conditions and **(B)** differences in transfer across experimental conditions. *p < 0.05, **p < 0.001, ***p < 0.001.

ANOVA showed that there was a significant difference across the three experimental conditions in retention [*F*(2,101) = 19.37, *p* < 0.001, $\eta_{p}^{2}$ = 0.28; see Figure 4A]. *Post hoc* tests (least significant difference; LSD) showed that the retention level of learners in the NDS group was significantly different from those in the TDS and UDS groups (MD = 3.06, *p* = 0.001, 95% CI [1.36, 4.75]; MD = 5.34, *p* < 0.001, 95% CI [3.63, 7.05]; respectively). The learners in the TDS group also showed better retention than the learners in the UDS group (MD = 2.29, *p* = 0.009, 95% CI [0.58, 4.00]). The results showed that the number of danmaku sent by learners while viewing the instructional video affected their learning retention.

ANOVA also revealed a significant difference across the three experimental conditions in transfer [*F*(2,101) = 6.75, *p* < 0.001, $\eta_{p}^{2}$ = 0.12; see Figure 4B]. *Post hoc* tests (LSD) showed that students in the NDS group had a better transfer level than those in the TDS and UDS groups (MD = 2.14, *p* = 0.037, 95% CI [0.13, 4.15]; MD = 3.73, *p* < 0.001, 95% CI [1.71, 5.76]; respectively). There was no significant difference between the TDS and UDS groups (*p* > 0.05). The results indicated that learners who did not send danmaku on the screen during video learning had the best transfer. Therefore, Hypothesis 1 was supported.

### Cognitive load

**FIGURE 5:**
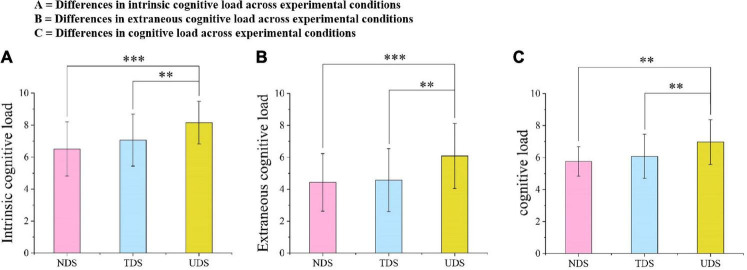
Differences in cognitive load across the experimental conditions. **(A)** Differences in intrinsic cognitive load across experimental conditions; **(B)** differences in extraneous cognitive load across experimental conditions; and **(C)** differences in cognitive load across experimental conditions. **p < 0.01, ***p < 0.001.

First, ANOVA found a significant difference in intrinsic cognitive load across the three experimental conditions [*F*(2,101) = 9.81, *p* < 0.001, $\eta_{p}^{2}$ = 0.16; see Figure 5A]. *Post hoc* tests (LSD) revealed that the intrinsic cognitive load of the UDS group was significantly different from that of both the TDS and NDS groups (MD = 1.09, *p* = 0.004, 95% CI [0.35, 1.83]; MD = 1.63 *p* < 0.001, 95% CI [0.89, 2.38]; respectively). Second, ANOVA showed that there was a significant difference in extraneous cognitive load across the experimental conditions [*F*(2,101) = 8.60, *p* < 0.001, $\eta_{p}^{2}$ = 0.15; see Figure 5B]. *Post hoc* tests (LSD) showed that the self-reported extraneous cognitive load of learners in the UDS group was significantly different from that in the TDS and NDS groups (MD = 1.52, *p* = 0.002, 95% CI [0.59, 2.44]; MD = 1.66, *p* = 0.001, 95% CI [0.73, 2.59]; respectively). However, no statistically significant difference was found compared to the ANOVA test on learners’ self-reported germane cognitive load. Finally, ANOVA also revealed a significant difference in cognitive load across the three experimental conditions [*F*(2,101) = 8.60, *p* < 0.001, $\eta_{p}^{2}$ = 0.15; see Figure 5C]. *Post hoc* tests (LSD) demonstrated that learners in the UDS group reported higher cognitive load than both the TDS and NDS groups (MD = 0.89, *p* = 0.004, 95% CI [0.30, 1.49]; MD = 1.21, *p* < 0.001, 95% CI [0.61, 1.81]; respectively). These self-report results suggest that sending danmaku onto the screen can influence learners’ cognitive load, as evidenced by the fact that learners in the UDS group had a higher cognitive load (including intrinsic and extraneous cognitive load) than reported by the learners in the other two groups. Thus, Hypothesis 2 was partially supported.

### Parasocial interaction

**FIGURE 6:**
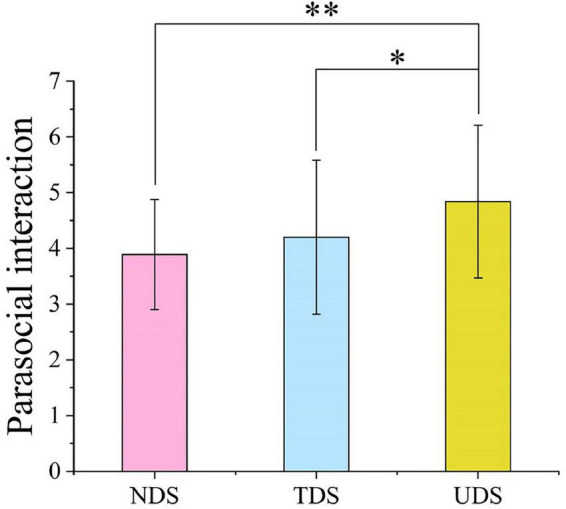
Differences in parasocial interaction across the experimental conditions. *p < 0.05, **p < 0.01.

ANOVA showed that there was a significant difference in learners’ parasocial interaction across the three experimental conditions [*F*(2,101) = 5.14, *p* = 0.007, $\eta_{p}^{2}$ = 0.09; see Figure 6]. *Post hoc* tests (LSD) revealed a statistically significant difference in the UDS group relative to the TDS (MD = 0.64, *p* = 0.036, 95% CI [0.04, 1.25]) and NDS (MD = 0.95, *p* = 0.002, 95% CI [0.35, 1.56]) groups. It was concluded that learners who sent danmaku on the screen with no limit to how many they could send had higher parasocial interaction scores compared to those of the learners in the other two groups. Thus, Hypothesis 3 was supported.

### Regression analysis and mediating effect

#### Intercorrelations among research variables

The Pearson correlation coefficients between the research variables and their significance levels are shown in Table 3. In terms of learning performance, learners’ knowledge retention scores were significantly negatively linked with conditions and parasocial interaction (*r*s = −0.53, −0.21, *p*s < 0.05). Knowledge transfer scores were significantly positively linked with retention scores (*r* = 0.54, *p* < 0.001), but significantly negatively linked with conditions (*r* = −0.34, *p* < 0.001). In terms of learning process experience, cognitive load was significantly positively linked with conditions, parasocial interaction, intrinsic, extraneous, and germane cognitive loads (0.37 < *r*s < 0.72, *p*s < 0.01). Extraneous cognitive load was significantly positively linked with conditions and intrinsic cognitive load (*r*s = 0.29, 0.33, *p*s < 0.01). Intrinsic cognitive load was significantly positively linked with conditions (*r* = 0.40, *p* < 0.001). Parasocial interaction was also significantly positively linked with conditions (*r* = 0.30, *p* = 0.002). There were no significant links among the remaining variables.

**TABLE 3:** Pearson correlations for research variables.

Variables	1	2	3	4	5	6	7
1. Conditions	–						
2. Parasocial interaction	0.30**	–					
3. Intrinsic cognitive load	0.40***	0.16	–				
4. Extraneous cognitive load	0.33**	0.17	0.29*	–			
5. Germane cognitive load	0.06	0.33**	0.17	0.26*	–		
6. Cognitive load	0.37***	0.33**	0.61***	0.72***	0.72***	–	
7. Retention	−0.53***	–0.04	−0.21*	–0.18	0.01	–0.18	–
8. Transfer	−0.34***	0.004	–0.11	0.01	0.17	0.05	0.54***

Pearson correlation coefficients and significance levels reported. ***p < 0.001, **p < 0.01, *p < 0.05.

#### Mediating effect analysis

**FIGURE 7:**
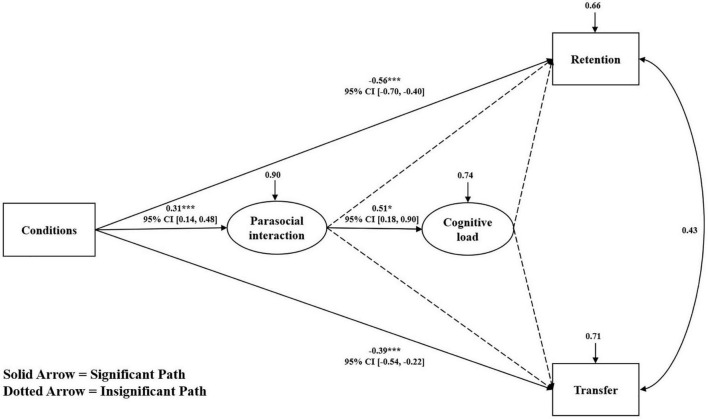
Mediation analysis with path coefficients. CI, confidence interval. *p < 0.05, ***p < 0.001.

The results of ANOVA showed that the retention and transfer scores of learners under different conditions were opposite to their self-reported cognitive load and parasocial interaction, and Pearson correlation analysis also found similar results. Therefore, this study constructed a mediation model (see Figure 7) to test whether the effects of the conditions on retention and transfer could be explained by parasocial interaction and cognitive load. The mediating effects were analyzed by standardizing all scores (*Z*-score) and performing structural equation modeling. To ensure conciseness in the model, all insignificant path coefficients and confidence intervals were deleted from the initial model. This model demonstrated a good data fit (χ^2^/*df* = 1.44, comparative fit index = 0.96; Tucker-Lewis index = 0.95, root mean square error of approximation = 0.07, standardized residual mean root = 0.08). Figure 7 shows the hypothesis testing results of the direct and indirect path coefficients of the mediation model. The results showed that all conditions had a significant direct effect on retention and transfer (β = −0.56, *p* < 0.001; β = −0.39, *p* < 0.001; respectively). Conditions not only significantly positively influenced parasocial interaction (β = 0.31, *p* < 0.001), but parasocial interaction also significantly positively influenced cognitive load (β = 0.31, *p* = 0.021). However, parasocial interaction and cognitive load did not influence retention and transfer (*p*s > 0.05). Therefore, Hypothesis 4 was not supported.

## Discussion

The primary result of this study was that learners in the NDS group scored higher on both retention and transfer than those in the TDS and UDS groups. However, learners’ self-reported cognitive load and parasocial interaction scores contradicted the finding on learning performance. There was also evidence that the more danmaku sent, the higher the learners’ level of parasocial interaction, which leads to a higher cognitive load. However, excessive parasocial interaction and cognitive load were not factors in the poor learning performance of learners. In the remainder of this discussion, we consider some of the differences between our findings and previous work on social interaction in learning, and then discuss some of the limitations of this evidence.

Our findings contrast with some reports in the existing literature that learners who participated in real-time exchange of messages in the classroom performed better in their learning (Weisz et al., 2007; Li et al., 2014). This study found that the more danmaku were sent, the less attentional impact they had on the learners. The survey data also indicated that only a small minority of learners preferred to send danmaku while learning. Indeed, a considerable number of learners believed that sending danmaku would hinder their learning ability. In their opinion, there were two main reasons for this expected negative impact: distraction and wasting time, both of which would greatly reduce their efficiency in learning. Learners did waste a lot of time on sending danmaku, ignoring the learning content itself, particularly the learners in the UDS group. The content of danmaku sent by the learners in the UDS group may have been random, too frequent, or dense, and may have also interfered with learners’ attention (Yang et al., 2019). Therefore, the more danmaku were sent, the easier it was to induce the split-attention effect (Ayres and Sweller, 2005). However, when learners interacted with others in the process of video learning, they had to be paying constant attention to and replying to the danmaku information scrolling along the top of the screen while also processing the learning content information. The learners had to integrate both the learning content and the danmaku information with their existing relevant knowledge, which led to the UDS group reporting a higher cognitive load in the process of video learning. In theory, higher cognitive load could lead to a lower learning performance (Ayres and Sweller, 2005; Yang et al., 2019). However, the mediation model constructed in this study did not support this assumption. Instead, it suggested that cognitive load was not an indirect cause of poor learning performance. Future research should further clarify the impact mechanism at play from the aspect of attentional impact. Furthermore, regarding the type of danmaku sent, some of the danmaku sent were social or novelty danmaku, which may have had little impact on the cognitive process of the learners. Regarding the learning content, the learning material was information about the heart. Learners needed to learn a great deal of knowledge in a short period of time, which may have been too challenging or difficult for the learners, making it easy to create a “floor effect” (Yang et al., 2019). Moreover, most of the learners in the UDS group continued to interact with each other in the form of danmaku. This phenomenon may have inhibited their creativity, as it has already been established that it is not conducive to the retention and transfer of new knowledge (Diehl and Stroebe, 1987).

Why did the UDS condition improve parasocial interaction? The purpose of learners sending danmaku was to cause them to interact more effectively with the others in their group, changing them from passive recipients to active participants in learning. One of the biggest drawbacks of video learning is its lack of social interaction, especially between fellow learners (Palaigeorgiou and Papadopoulou, 2019). By sending danmaku, the learners were given the illusion of being connected in a digital world, as if they were communicating in real-time with others. In addition, the interactivity of sending danmaku is related to the cognitive process of learners (Zhang et al., 2019). Looking at the level or degree of learner interaction from the group with the lowest (NDS) to the highest (UDS) amount of communication reflected the learners’ degree of mental engagement and the fact that the learners are guided into being more active participants (Patwardhan and Murthy, 2015). Vorderer et al. (2004) viewed parasocial interaction as a core precondition for media enjoyment and stated that this phenomenon facilitates learners’ subsequent engagement with their respective content. Thus, stronger parasocial interaction seem to promote better learning performance in an instructional video settings (Beege et al., 2017). Parasocial interactions did not appear to be directly linked to learning performance in the present findings. Future work should therefore also investigate other variables such as arousal, affective, or motivational variables to gain insight into the underlying variables influencing learning performance (Beege et al., 2017). Interestingly, the mediation model constructed in this study showed that parasocial interaction increased cognitive load, making it clear that parasocial interaction has an impact on cognitive processes. Therefore, future studies should also provide more empirical evidence of this. Cognitive load theory and cognitive theory of multimedia learning have both been introduced as ways to expand the understanding of parasocial interaction (Ayres and Sweller, 2005; Mayer, 2005).

More generally, what stood out in the current study was that sending danmaku while learning from instructional videos not only hindered learners’ learning performance, but also, the more danmaku sent, the greater the negative impact on learning. Although this type of social interaction can effectively improve learners’ level of parasocial interaction, it can also increase their cognitive load.

The findings of the current study lead to some practical advice for instructional video designers, specifically, that danmaku have the characteristics of timely feedback and strong interaction, and it can meet the personalized learning needs of learners and improve their learning experience (Yang et al., 2019). However, if instructors want their learners to achieve better learning performance, they should not ask learners to send danmaku while the instructional videos are in progress. Therefore, if instructors want learners to have an interactive learning experience, we suggest that these interactions should take place after students are finished learning from the instructional videos (Pi et al., 2020).

Three features of this work limit the conclusions we can draw about the effects of learners sending danmaku in the process of their learning. First, this study only measured behavioral data which reflected the learning process of learners, and it is unclear whether other more objective and reasonable measurement techniques would produce comparable results. Future research may consider introducing eye-tracking and fMRI techniques to further reveal the cognitive and neural mechanisms of the impact of sending danmaku on learners’ learning from an interdisciplinary perspective. Specifically, portable eye-tracking technology has been used to explore whether variables such as learners’ preference for danmaku sending or danmaku style would have a moderating effect. fMRI and physiological polygraph have been used to measure blood flow changes caused by learners’ neuron activity and the changes they experience in academic emotions at the physiological level, to seek out reasons that can be explained from a neurological and physiological perspective (Guo et al., 2019). Second, the findings of this study should not be generalized beyond the high school student population or other populations with similar demographic characteristics. In future studies, a wider range of data from across different disciplines should be collected from more participants from different schools and at different age levels (Wu et al., 2021). Finally, knowledge type may be an important moderator variable in the learning experience. In terms of declarative knowledge, danmaku did not play a role in improving this. However, for procedural knowledge, danmaku can in fact improve knowledge transfer (Yang et al., 2019). Therefore, future work can start from an understanding of different knowledge types in order to conduct further experiments.

## Data availability statement

The original contributions presented in this study are included in the article/supplementary material, further inquiries can be directed to the corresponding author.

## Author contributions

CW contributed to the study conception and design. BJ, YL, and NF performed the material preparation and data collection. BJ and YM performed the data analysis and wrote the first draft of the manuscript. All authors commented on previous versions of the manuscript, read, and approved the final manuscript.

## Footnotes

1
www.dandanplay.com
